# Targeting RNA‐binding motif protein 39 for arginine reduction: unveiling metabolic vulnerability in arginine‐dependent liver cancer

**DOI:** 10.1002/mco2.581

**Published:** 2024-06-19

**Authors:** Aoxue Li, Hongjuan Cui, Erhu Zhao

**Affiliations:** ^1^ State Key Laboratory of Resource Insects, Medical Research Institute, Southwest University Chongqing China; ^2^ Jinfeng Laboratory Chongqing China; ^3^ Chongqing Engineering and Technology Research Center for Silk Biomaterials and Regenerative Medicine Chongqing China

**Keywords:** amino acid metabolism, cancer biology, metabolic reprogramming

## Abstract

Cancer is increasingly acknowledged as a metabolic disease, characterized by metabolic reprogramming as its hallmark. However, the precise mechanisms behind this phenomenon and the factors contributing to tumorigenicity are still poorly understood. In a recent publication in *Cell*, Mossmann and colleague reported a study unveiling arginine as a molecule with second messenger‐like properties that reshapes metabolism to facilitate the tumor development in hepatocellular carcinoma (HCC). Their research revealed that the RNA‐binding motif protein 39 (RBM39)‐mediated increase in asparagine synthesis results in increased arginine uptake. This establishes a positive feedback loop that sustains elevated levels of arginine and facilitates oncogenic metabolic reprogramming. Additionally, Mossmann et al. demonstrated that depleting RBM39 with indisulam effectively disrupts the proto‐oncogenic metabolic reprogramming in HCC. This discovery presents a novel treatment strategy for arginine‐dependent liver cancers.

## Graphical abstract

In a recent publication in *Cell*, Mossmann and colleagues presented a study uncovering arginine's role as a molecule with characteristics akin to second messengers, reshaping metabolism to promote tumor growth in hepatocellular carcinoma (HCC). Their investigation demonstrated that the RNA‐binding motif protein 39 (RBM39) drives heightened asparagine synthesis, leading to elevated arginine absorption. This sets up a reinforcing cycle that supports sustained arginine levels and promotes oncogenic metabolic alterations. This finding introduces a fresh therapeutic approach for arginine‐dependent liver malignancies.

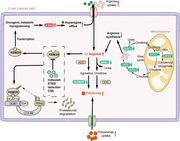

1

Cancer is increasingly acknowledged as a metabolic disease, characterized by metabolic reprogramming as its hallmark.^1^ However, the precise mechanisms behind this phenomenon and the factors contributing to tumorigenicity are still poorly understood. In a recent publication in *Cell*, Mossmann and colleague reported a study unveiling arginine as a molecule with second messenger‐like properties that reshapes metabolism to facilitate the tumor development in hepatocellular carcinoma (HCC).^2^ Their research revealed that the RNA‐binding motif protein 39 (RBM39)‐mediated increase in asparagine synthesis results in increased arginine uptake. This establishes a positive feedback loop that sustains elevated levels of arginine and facilitates oncogenic metabolic reprogramming. Additionally, Mossmann et al.^2^ demonstrated that depleting RBM39 with indisulam effectively disrupts the proto‐oncogenic metabolic reprogramming in HCC (Figure 1). This discovery presents a novel treatment strategy for arginine‐dependent liver cancers.

**FIGURE 1:**
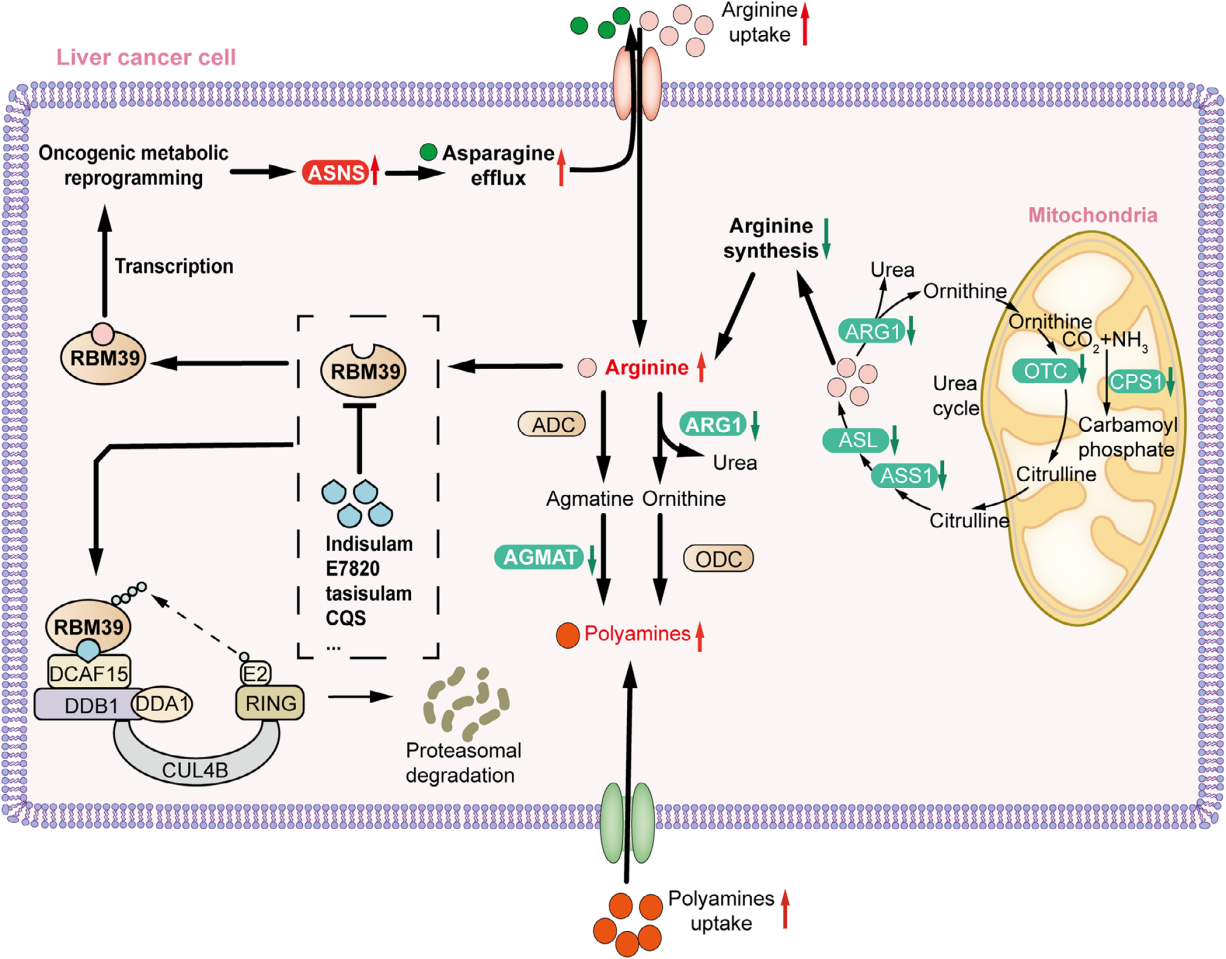
Model for arginine‐driven oncogenic metabolic reprogramming in HCC cells. Red arrows indicate elevated levels of metabolites or enzyme activity in tumor cells compared to normal liver cells, while green arrows denote the opposite. ADC, arginine decarboxylase; ASL, argininosuccinate lyase; CPS1, carbamoyl phosphate synthetase 1; CUL4B, cullin 4B; DCAF15, DDB1 and CUL4 associated factor 15; DDA1, DET1 and DDB1 associated 1; DDB1, damage specific DNA binding protein 1; HCC, hepatocellular carcinoma; OTC, ornithine transcarba‐mylase; ODC, Ornithine decarboxylase.

Arginine serves a wide range of functions, participating in protein synthesis and interconverting with proline and glutamate. Moreover, it is able to stimulate cell vitality through the activation of mTORC1.^3^ The primary pathway for endogenous synthesis of arginine is through the urea cycle, with arginine succinate synthase 1 (ASS1) serving as the rate‐limiting enzyme in arginine synthesis. Interestingly, Mossmann et al. observed a decrease in the expression of arginine synthesis genes in mice and in patients with HCC, despite elevated arginine levels. Subsequently, Mossmann et al. conducted a comprehensive analysis of tumor samples from both mouse models and patients, utilizing off‐target metabolomics, transcriptomics, and proteomics to gain insight into the oncogenic metabolic alterations. The findings indicate inhibition of the urea cycle in arginine metabolism, along with minimal expression of genes associated with arginine synthesis, despite the elevated concentrations of arginine observed within the tumor. Mossmann et al. also elucidated the early inhibition of two enzymes, arginase 1 (ARG1) and agmatinase (AGMAT), responsible for converting arginine into polyamines. Additionally, they observed the transcriptional upregulation of several transporter proteins from the solute carrier 7A family (SLC7A1, SLC7A3, SLC7A4, SLC7A6, SLC7A7, and SLC7A9) involved in the uptake of arginine, indicating a compensatory increase in the uptake of arginine to counterbalance the reduced arginine synthesis in tumors.

ARG1 and AGMAT facilitate the conversion of arginine into polyamines through two parallel pathways (Figure 1). The reduced conversion of arginine to polyamines results in elevated levels of arginine in tumors, a situation maintained by the low expression of ARG1 and AGMAT. Polyamines play a critical role in cell growth and are found at elevated levels in various types of cancers. Despite the reduction of ARG1 and AGMAT, polyamine levels remain elevated in cancer. Experiments involving arginine‐restricted diets have indicated that the increased polyamine levels are not solely attributable to endogenous uptake from arginine accumulation.^2^ However, the precise cause of the elevated polyamine levels in cancer remains to be fully understood.

Tumor cells with elevated arginine levels exhibit significant upregulation of asparagine synthetase (ASNS), phosphoserine aminotransferase 1 (PSAT1), phosphoserine phosphatase (PSPH), and glutaminase kidney isoform (GLSK), while glucose transporter 3 (GLUT3), hexokinase 2 (HK2), NAD^+^ metabolic gene nicotinamide N‐methyltransferase (NNMT), and primary amine oxidase 3 (AOC3) show an inverse pattern of expression. Among these genes, ASNS exhibited the most pronounced differential expression. Asparagine has been identified as an amino acid exchange factor that influences the uptake of various extracellular amino acids, particularly arginine. Mossmann et al. further confirmed that ASNA promotes arginine uptake into tumor cells and is crucial for the tumor formation in vivo. Importantly, the reduced expression of ARG1 and AGMAT contributes to the accumulation of arginine, while the ASNS expression dependent on arginine leads to asparagine generation, thereby enhancing arginine uptake. This establishes a positive feedback loop that maintains the high level of arginine in tumor cells.^2^

RBM39 functions as an RNA‐binding protein engaged in transcriptional coregulation and selective RNA splicing. It comprises an N‐terminal serine‐rich‐serine (RS) domain, two RNA recognition motif (RRM1/2) domains, and a C‐terminal U2AF homology motif (UHM) domain. Mossmann et al. discovered that RBM39 is most likely to bind arginine at the N‐terminus of the RRM1 domain. Additionally, it was found that RBM39 regulates the metabolic gene expressions through transcription instead of pre‐mRNA splicing. The transcriptional control of ASNA expression by RBM39 results in increased arginine uptake, thereby further enhancing tumorigenicity and promoting the tumor development. ASNS and RBM39 exhibit the elevated expression in embryonic liver and reduced expression in adult liver.^2^ This pattern reflects their role in regulating tumor metabolic reprogramming and suggests that they may activate tumor cells to revert to an undifferentiated embryonic metabolic state.

Furthermore, RBM39 serves as a novel innate immunity factor with extensive effects on innate immunity by regulating the transcription and/or selective splicing of interferon regulatory factor 3 (IRF3) and other essential proteins associated with the innate immune response. Arginine also serves a pivotal function in immune cells, including survival, proliferation, and activation of T‐cell, all of which are reliant on arginine. The significant consumption of arginine from the tumor microenvironment facilitates immune evasion in tumor cells. However, the strategy of inhibiting the tumor development by reducing arginine levels can inadvertently affect the normal functioning of immune cells. To address this concern, Mossmann et al. proposed targeting RBM39, which specifically binds to arginine, as a potential approach for tumor suppression that avoids compromising the immune cell function.

RBM39 is considered a target for sulfonamides, such as indisulam, E7820, tasisulam, and CQS, which have been demonstrated to induce the degradation of RBM39. For instance, indisulam can function as a molecular binder between RBM39 and DCAF15‐associated E3 ubiquitin ligase complex, resulting in targeted selective degradation.^4^ Moreover, the regulation of RBM39 by indisulam has been found to activate anti‐tumor immunity and enhance checkpoint immunotherapy.^5^ In the study by Mossmann et al., 16‐week‐old HCC model mice were administered seven injections of indisulam over a two‐week period. This treatment regimen effectively decreased the tumor development with no alteration in the liver‐to‐body weight ratio. Further investigations in cells, mice, and patient‐derived organoids have shown potential for aryl sulfonamides analogs in treating HCC patients with elevated levels of arginine and RBM39.

In summary, Mossmann et al. illustrated how arginine functions as a molecule possessing second messenger‐like attributes that facilitates the tumor development by modulating the expression of metabolic gene through its specific interaction with RBM39. However, further investigation is needed to establish the exact binding site of RBM39 and arginine, the detailed mechanism of arginine binding to RBM39, and the transcription factors that facilitate the metabolic gene expression influenced by RBM39 (arginine‐dependent effects). Additionally, a significant arginine metabolism pathway, known as the nitric oxide pathway, involves nitric oxide synthase 2 (NOS2) in the production of nitric oxide (NO) and citrulline. Mossmann et al. revealed through RNA‐sequencing that RBM39 depletion did not affect the expression of NOS2 in liver cancer cells. However, it remains unclear whether the increased uptake of extracellular arginine is utilized by the NOS2 pathway and whether NOS2 contributes to the production of NO, potentially promoting the progression of HCC. These inquiries warrant further exploration. Moreover, arginine plays a pivotal role in modulating immune responses and significantly affects various metabolic pathways in immune cell biology. Drugs targeting RBM39, which selectively reduce arginine levels, may serve as immunosuppressants for individuals undergoing the treatment. Therefore, focusing on targeting arginine to regulate tumor metabolism may offer an effective and less toxic strategy to improve the prognosis of arginine‐dependent liver cancer patients.

## AUTHOR CONTRIBUTIONS

Aoxue Li wrote the manuscripts. Erhu Zhao and Hongjuan Cui reviewed and modified the manuscript. All authors have read and approved the final manuscript.

## CONFLICT OF INTEREST STATEMENT

The authors declare no conflicts of interest.

## ETHICS STATEMENT

 No ethical approval was necessary for this work.

2

## Data Availability

No data was used for the research described in this work.
